# Using a Mobile Phone App to Analyze the Relationship Between Planned and Performed Physical Activity in University Students: Observational Study

**DOI:** 10.2196/17581

**Published:** 2021-04-29

**Authors:** Matthew T Stewart, Taylor Nezich, Joyce M Lee, Rebecca E Hasson, Natalie Colabianchi

**Affiliations:** 1 School of Kinesiology University of Michigan Ann Arbor, MI United States; 2 Susan B. Meister Child Health Evaluation and Research Center Department of Pediatrics University of Michigan Medical School Ann Arbor, MI United States; 3 Department of Nutritional Sciences School of Public Health University of Michigan Ann Arbor, MI United States; 4 Institute for Social Research University of Michigan Ann Arbor, MI United States

**Keywords:** mobile phone application, physical activity, intention-behavior relationship

## Abstract

**Background:**

The relationship between intention and behavior has been well researched, but most studies fail to capture dynamic, time-varying contextual factors. Ecological momentary assessment through mobile phone technology is an innovative method for collecting data in real time, including time-use data. However, only a limited number of studies have examined day-level plans to be physically active and subsequent physical activity behavior using real-time time-use data to better understand this relationship.

**Objective:**

This study aims to examine whether plans to be physically active (recorded in advance on an electronic calendar) were associated with objectively assessed physical activity (accelerometry), to identify activities that replaced planned periods of physical activity by using the mobile app Life in a Day (LIAD), and to test the feasibility and acceptability of LIAD for collecting real-time time-use data.

**Methods:**

The study included 48 university students who were randomly assigned to 1 of 3 protocols, which were defined by 1, 3, or 5 days of data collection. Participants were asked to record their planned activities on a Google Calendar and were provided with mobile phones with LIAD to complete time-use entries in real time for a set of categories (eg, exercise or sports, eating or cooking, school, or personal care). Participants were instructed to wear an accelerometer on their nondominant wrist during the protocol period. A total of 144 days of protocol data were collected from the 48 participants.

**Results:**

Protocol data for 123 days were eligible for analysis. A Fisher exact test showed a statistically significant association between plans and physical activity behavior (*P*=.02). The congruence between plans and behavior was fair (Cohen κ=0.220; 95% CI 0.028-0.411). Most participants did not plan to be active, which occurred on 75.6% (93/123) of days. Of these 93 days, no physical activity occurred on 76 (81.7%) days, whereas some physical activity occurred on 17 (18.3%) days. On the remaining 24.4% (30/123) of days, some physical activity was planned. Of these 30 days, no physical activity occurred on 18 (60%) days, whereas some physical activity occurred on 12 (40%) days. LIAD data indicated that activities related to screen time most often replaced planned physical activity, whereas unplanned physical activity was often related to active transport. Feasibility analyses indicated little difficulty in using LIAD, and there were no significant differences in feasibility by protocol length.

**Conclusions:**

Consistent with previous literature, physical activity plans and physical activity behaviors were linked, but not strongly linked. LIAD offers insight into the relationship between plans and behavior, highlighting the importance of active transport for physical activity and the influence of screen-related behaviors on insufficient physical activity. LIAD is a feasible and practical method for collecting time-use data in real time.

## Introduction

### Background

The relationship between physical activity, chronic disease morbidity, and all-cause mortality is well documented. In adults, consistent physical activity can help mitigate risk factors for chronic diseases, including cardiovascular disease, diabetes, cancer, hypertension, obesity, and depression [1]. Although these benefits are widely recognized, approximately 77% of US adults do not meet the current recommendations for aerobic and muscle-strengthening activities [2]. Moreover, marked declines in physical activity participation begin in adolescence, a trend that is sustained in older adulthood [3,4]. Life-transition events, characterized by a significant disruption in previously established routines and habits, can exacerbate these declines [5,6]. One such group experiencing a transitional event is university students, who often cite a newfound lack of time or motivation to be active, subsequently failing to meet the current physical activity guidelines and developing patterns of inactivity that may persist into adulthood [7-9].

Contemporary models suggest that physical activity behavior arises from the interplay of individual, societal, and environmental characteristics [10]. A classic theoretical framework, the theory of planned behavior (TPB), posits that physical activity behavior is predicted by the intention to be active [11,12]. The relationship between intention and behavior has been widely demonstrated in the literature, and as such, the TPB remains a prevalent theoretical framework in physical activity behavior research [13]. However, the strength of the intention-behavior relationship is not consistently robust [14,15]. This inconsistency often stems from the divergence of positive intenders: those who intend to be active but do not act [16]. Research using the TPB has found that those who intend to perform a behavior are more likely to engage in their intended behavior if they make a *plan* to carry out their intentions, including when they will perform their behavior [17].

Recent criticisms of the TPB suggest that the gap between intention and behavior may emerge from the TPB’s failure to include the effect of time and context, instead viewing physical activity as a static behavior [18]. Consequently, there is limited evidence regarding *day-level* intentions to be active and subsequent physical activity. A promising method for collecting data in real time is ecological momentary assessment (EMA). This method involves the repeated measurement of current behaviors and contextual factors, often by way of using mobile phones, while minimizing the bias found in methods that require recall well after the activity has occurred [19-21]. The literature regarding EMA has suggested that momentary intentions positively predict subsequent physical activity in the hours following an EMA prompt [22,23]. However, it is not clear whether this is the appropriate time scale, as engaging in physical activity may have logistical issues (eg, finding available time or being able to get to the location where the activity will be performed), which may need to be considered further in advance. These logistical issues may make having a plan to be active particularly predictive of subsequent physical activity behavior. Furthermore, although the continuous monitoring of EMA has advantages when capturing dynamic, time-varying changes in intention and plans and when predicting behavior, certain EMA features, such as excessive prompting or text messaging, may limit engagement and hinder feasibility [24,25]. When prompting, EMAs often use signal-contingent sampling (sampling based on fixed or random times) or context-aware sampling (sampling based on events defined by automatic sensing technologies), which may interrupt activities when they are occurring and hinder compliance. Conversely, time-use diaries using event-contingent recording may avoid this distraction by asking participants to record information when an event begins and ends [26-28].

### Objectives

Therefore, the purpose of this study is three-fold. The first aim is to examine whether plans to be physically active, recorded in advance on an electronic Google Calendar, were associated with objectively assessed (Actigraph GT9X Link) physical activity over a period of 1, 3, or 5 days. The second aim is to use data from a time-use mobile app (Life in a Day [LIAD]) using event-contingent recording to identify what pursuits occurred when participants had planned to be active but were not and when unplanned physical activity occurred. The final aim is to test the feasibility and acceptability of LIAD for collecting real-time time-use data among university students across 3 different protocol lengths.

## Methods

### Overview

Several recruitment methods were employed. The primary recruitment method was flyers posted in university student spaces detailing information about participation in the study. Researchers also used direct, face-to-face contact with potential participants around campus to aid with recruitment. Participants could inform others of the study, although no compensation was provided to them for their recruitment efforts. Participants recruited via word of mouth from other participants were required to contact us to participate in the study. The subject population was limited to enrolled undergraduates at the University of Michigan aged between 18 years and 25 years. Participants also had to be planning to remain in the area for the 2 weeks following enrollment and be comfortable with using the provided mobile phone and accelerometer. The base compensation was US $20 for the 1-day protocol, which increased for the 3- and 5-day protocols. Written informed consent was obtained from each participant, detailing their voluntary participation in a study that used mobile devices to collect information regarding time use and physical activity. This study was approved by the institutional review board of the University of Michigan.

Data collection began in the summer and ended early in the winter semester, from late July to early February. Breaks in data collection were periodic to avoid scheduled university holidays and examinations, including an extended winter break from early December to mid-January. Participants completed an initial intake meeting, at which they completed an introductory demographic survey consisting of questions regarding age, sex, race, parent education, and engagement in common phone activities (eg, texting, calling, or social media browsing). Participants also received an accelerometer and a mobile phone. The study phone was provided to limit technological issues based on different phone types and potentially limited data availability when using a participant’s phone. After providing written informed consent, participants were trained on how to wear the accelerometer and use LIAD (eg, given an explanation of the 12 categories of activities) and were shown how to record physical activity plans and other activities on their Google Calendar. Participants gave verbal confirmation that they understood the protocol and were also given take-home instructions with contact information should they have any follow-up questions. Participants were randomly assigned to a 1-, 3-, or 5-day protocol at this intake meeting. Three different protocol lengths were used to highlight differences in feasibility. Participants were asked to carry their mobile phone with them and wear the accelerometer for the full duration of the study.

**Figure 1:**
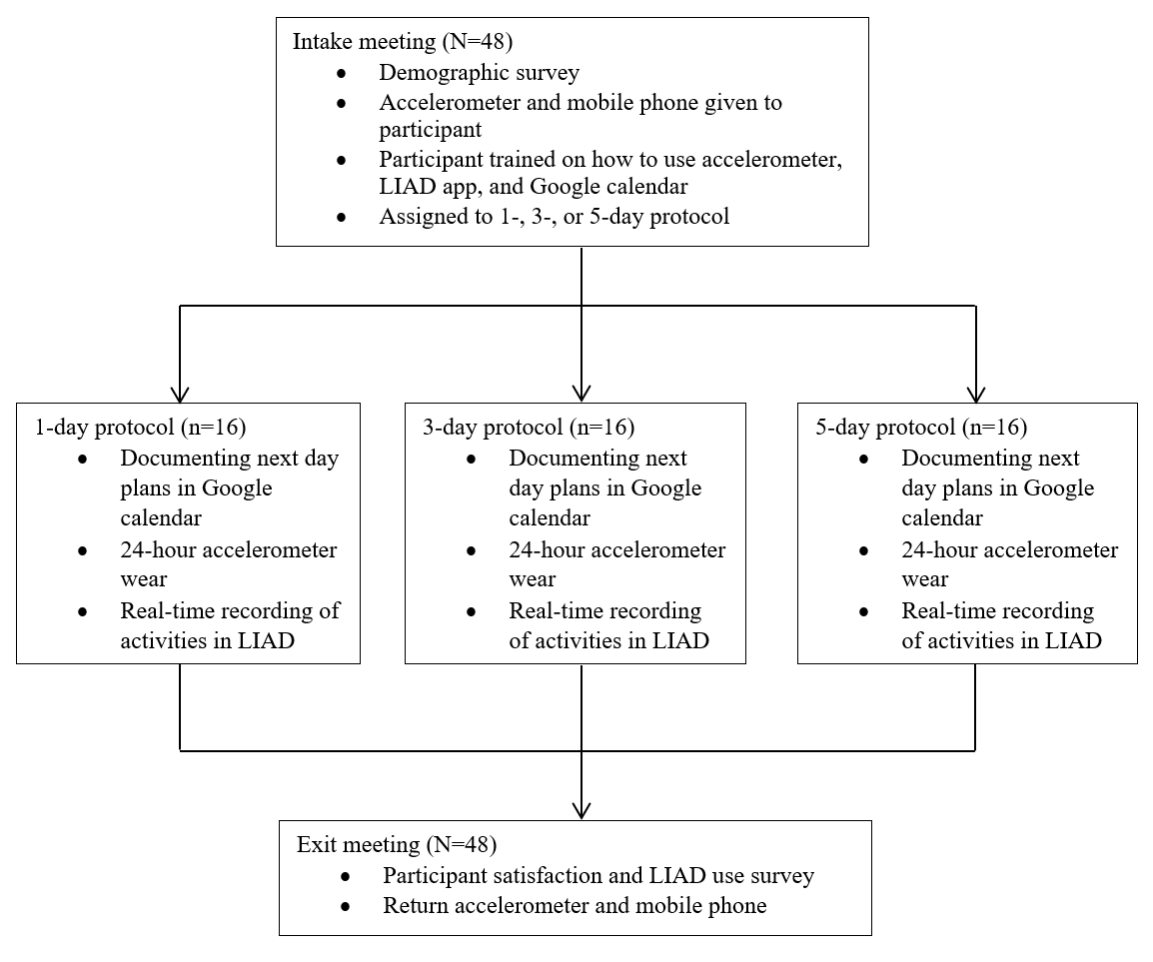
Study flow. LIAD: Life in a Day.

Participants were asked to go about their lives as usual. Before each day of the protocol, participants were asked to record activities planned for the next day on a separate Google Calendar. Throughout each day of the protocol, participants were asked to record activities in real time on LIAD. Activities were filled in retrospectively if they were not completed in real time. At the end of the protocol period, participants returned the study equipment and completed an exit survey, which consisted of questions regarding participant satisfaction, LIAD ease of use and recording in real time, and any issues that may have hindered compliance or engagement. No phone or accelerometer was lost. Once data were downloaded from the mobile phone, the data were transferred to a secure, password-protected server. After transfer, any remaining participant data on the mobile phone were erased. The study flow is shown in Figure 1.

### Physical Activity

Physical activity was objectively measured via accelerometry using an Actigraph GT9X Link. Accelerometry is a valid method for capturing physical activity behavior [29,30]. Participants were asked to wear the accelerometer on their nondominant wrist for 24 hours per day for the full protocol period. Each accelerometer was initialized to collect data at 80 Hz (ie, 80 observations per second) for each of the 3 axes. Raw accelerometer data in units of gravity were analyzed using the R package GGIR version 1.8-1, a widely tested software package for quantifying physical activity data across numerous brands of monitors [31-35]. Nonwear was determined by an automated algorithm in GGIR that classifies time as nonwear when the SD is less than 13 mg for 2 of the 3 axes or if the value of each accelerometer axis is less than 150 mg, which is determined using moving windows of 60 minutes within 15-minute increments [32]. A minimum of 10 hours of wear time was required to include that day in the analyses. An acceleration threshold of 200 mg was used to indicate moderate-to-vigorous physical activity (MVPA) based on the work of Hildebrand et al [36]. Data were analyzed using a 10-minute bout metric in which at least 80% of the bout needed to be at or above the MVPA threshold. Minutes of MVPA were calculated for each hour. These files were then merged with Google Calendar and LIAD data in Microsoft Excel to facilitate comparison.

### LIAD App

Activities performed throughout the day were self-tracked using an electronic time-use diary. Time-use diaries are the most common way to assess time use and are a valid method for capturing daily activities [28,37,38]. Electronic methods (eg, web- and app-based modes) were found to produce better quality data and a comparable number of logged activities compared with the paper method [39]. Each participant was provided with a mobile phone (Samsung Galaxy S5) with LIAD downloaded for the duration of the study. LIAD was developed by the Division of Cancer Control and Population Sciences at the US National Cancer Institute in collaboration with MEI Research Ltd and was previously found to be an easy-to-use and acceptable method of measuring time use [40]. The app allowed participants to self-track activities performed throughout the day into 1 of 12 categories: sleeping or doing nothing; personal care; eating or cooking; computer, television, or reading; household activities; entertainment or social activities; school; shopping, appointments, or errands; transport; work (for pay); exercise and sports; and nonsport organized activity. The research team chose these categories based on a review of categories used in the American Time Use Survey, the Panel Study of Income Dynamics (PSID), the Supplement on Disability and Use of Time, and the PSID Childhood Development Supplement [41-43]. If a participant’s activity did not fit into one of those categories, participants could add their own category. Participants could also record multiple activities simultaneously (eg, studying while eating dinner). Activities were tracked on LIAD by pressing a *Start* button when the activity began and a *Stop* button when the activity ended (Multimedia Appendices 1-3). Once an activity was recorded, it was automatically added to a calendar built in the app, which participants could view. Though encouraged to record activities in real time, participants could adjust start and stop times if needed or add activities into gaps within their calendar. Gaps were easily identified by viewing the LIAD calendar, which was accessible via the study mobile phone. LIAD was used to identify the activity that occurred if there was a discrepancy between what was planned on the Google Calendar and what was captured by the accelerometer. For a day to be eligible for analysis, participants needed at least three recorded activities on LIAD, which was considered the minimum number of entries that would occur on a single day.

### Google Calendar

Before each day of the protocol, participants were asked to record their planned activities for the following day on a Google Calendar. Other studies have successfully used calendars to document planned activities, particularly physical activity [44,45]. Unique email addresses and passwords were generated by the research staff and provided to each participant. Instructions were provided to the participants regarding how to fill out the calendar. Participants could either use the web-based Google Calendar or the mobile phone–based app. During data analysis, participants’ planned activities were classified into the same categories as those within LIAD to facilitate comparison. If any calendar data were present on a certain day (ie, if the participant recorded at least one planned activity via Google Calendar), that day was included in the analyses as it signified the use of the calendar feature.

### Feasibility

The primary indicators of feasibility included measures of accelerometer wear time, participants’ use of LIAD and Google Calendar, and the assessment of any documented participant issues. Accelerometer wear time was measured at the day level for each participant. Participants were asked feasibility questions at the completion of the study, including how easy it was to record activities on LIAD, their estimation of how often they were able to record activities in real time, any issues they may have encountered while recording activities in real time, and willingness to participate in a similar study again.

### Analysis

Demographic characteristics collected from the sample included age, sex (male or female), race (White, Black or African American, Asian, American Indian or Alaskan Native, Native Hawaiian or Pacific Islander, or other), parental education (high school degree, General Educational Diploma, or less; 2-year college or vocational school; 4-year college degree; or graduate degree), and common phone activities (eg, texting, voice calls, video calls, social media browsing, games, maps, directions, and shopping). To assess the congruence between planned and performed physical activity, 4 types of days were calculated: days where physical activity was planned *and* executed, planned *but not* executed, not planned *and* executed, and not planned *or* executed. The Cohen κ and Fisher exact test were used to summarize the congruence between planned and performed physical activity. Participants were categorized as having *planned physical activity* when they had an entry in their Google Calendar that specified an activity that would be physical activity. To determine whether any physical activity was executed on a given day, we used the accelerometry data, specifically a 10-minute bout of MVPA. Days with no plans for physical activity (according to the Google Calendar) were classified as having no planned physical activity. On days with no plans for physical activity, if any period met our 10-minute threshold for MVPA, we defined it as not planned *and* executed (ie, unplanned physical activity). If a participant planned physical activity on a certain day, we considered that plan to be executed if MVPA occurred at any point during that day for at least 10 minutes, regardless of timing or whether the activity lasted as long as proposed in the calendar. We conducted further analyses to assess the congruent timing of plans and MVPA (ie, executed MVPA that occurred during the same hour as planned in the Google Calendar). With respect to feasibility, compliance with each of the 3 protocol elements was described (accelerometer wear time, Google Calendar entries, and LIAD use). Fisher exact tests were then conducted to determine whether accelerometer wear time, difficulty using LIAD, reporting activities in real time, and completing Google Calendar varied by protocol length. A thematic analysis of each open-ended feasibility question and participant-reported issues was conducted.

## Results

### Overview

In total, there were 48 participants split evenly across the 3 protocol lengths (16 in each). Table 1 presents the demographic characteristics of the sample. For a day to be eligible for analysis, participants needed to have sufficient accelerometry data, LIAD data, and Google Calendar data (sufficient data are defined in the *Methods* section). Of the missing data, 2% (1/48) of participants had no accelerometry data (on the 5-day protocol), expressing during the exit survey that they had forgotten to wear the monitor. Less than 10 hours of valid accelerometer wear time occurred for at least one day for 15% (7/48) of participants. There were missing calendar data on at least one day for 8% (4/48) of participants, all expressing that they had forgotten to complete the calendar the night before. Each participant recorded at least three activities each day on LIAD. Thus, there were missing data across the protocol components for 14.6% (21/144) of days, leaving a total of 85.4% (123/144) of days eligible for analyses.

**Table 1:** Demographic characteristics of the study participants (N=48).

Characteristics		Values
Age (years), mean (SD)		19.8 (1.8)
**Sex, n (%)**		
	Female	37 (77)
	Male	11 (23)
**Race, n (%)**		
	White	17 (35)
	Black or African American	8 (17)
	Asian	19 (40)
	Other or multiple races	4 (8)
**Parent education: mother, n (%)**		
	High school, GED^a^, or less	11 (23)
	2-year or vocational school	6 (12)
	4-year college degree	21 (44)
	Graduate degree (eg, Master’s, JD^b^, MD^c^, or PhD^d^)	10 (21)
**Parent education: father, n (%)**		
	High school, GED, or less	8 (17)
	2-year or vocational school	3 (6)
	4-year college degree	17 (35)
	Graduate degree (eg, Master’s, JD, MD, or PhD)	20 (42)
**Common phone activities (multiple responses), n (%)**		
	Texting	48 (100)
	Voice calls	44 (92)
	Video calls	27 (56)
	Social media browsing	46 (96)
	Maps	41 (85)
	Directions	42 (88)
	Shopping	16 (33)
	Games	19 (40)
	Other	13 (27)
MVPA^e^ minutes, mean (SD)		4.8 (12.3)

^a^GED: General Educational Diploma.

^b^JD: Doctor of Law.

^c^MD: Doctor of Medicine.

^d^PhD: Doctor of Philosophy.

^e^MVPA: moderate-to-vigorous physical activity.

### Planned Versus Performed Physical Activity

**Figure 2:**
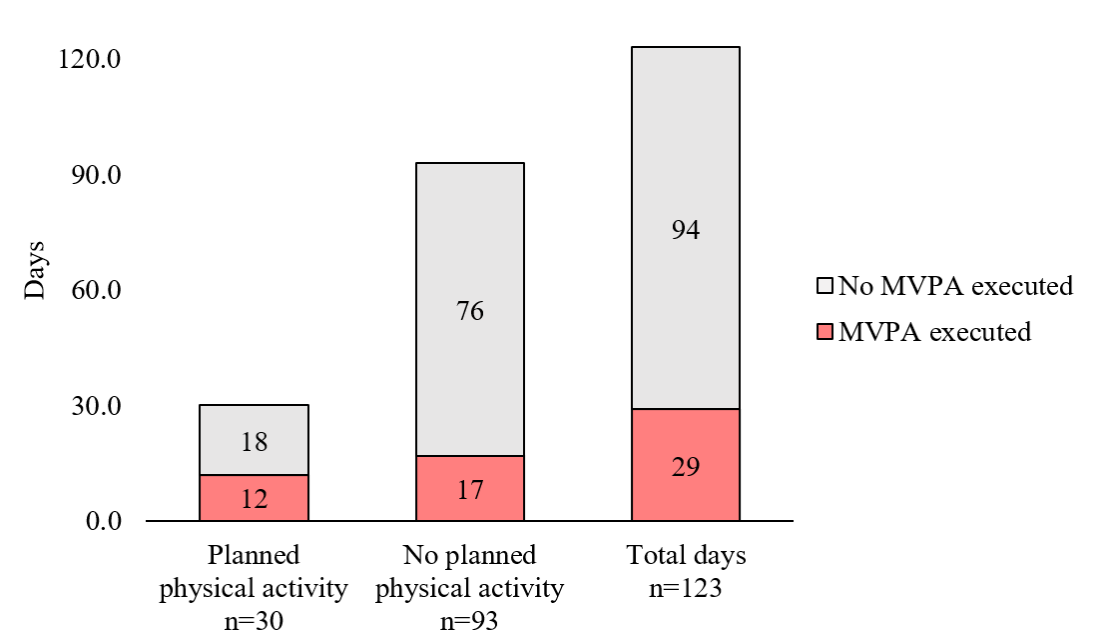
Planned versus performed physical activity. MVPA: moderate-to-vigorous physical activity.

The analysis of planned versus performed physical activity indicated that most participants did not plan any physical activity on their Google Calendars or participate in any physical activity, as shown by accelerometer data. Most participants did not plan to be active, which occurred on 75.6% (93/123) of days. Of those 93 days, no physical activity occurred on 76 (81.7%) days, whereas some physical activity occurred on 17 (18.3%) days. On the remaining 24.4% (30/123) of days, some physical activity was planned. Of those 30 days, no physical activity occurred on 18 (60%) days, whereas some physical activity occurred on 12 (40%) days. The Fisher exact test showed a statistically significant association between plans and physical activity behavior (*P*=.02). However, when assessing the degree of agreement between plans (or lack of plans) to be active and subsequent behavior using the Cohen κ, only fair agreement was found (κ=0.220; 95% CI 0.028-0.411). Although the popular nomenclature for interpreting κ values would suggest fair agreement, it is important to note that the implementation rate of physical activity among those who planned to be active was only 40% [46]. When requiring the accelerometer-derived physical activity to occur during the exact same hour as when the physical activity was planned, only 25% (3/12) of days had congruent periods of planned physical activity and MVPA, whereas MVPA was performed outside of the planned period on the remaining 75% (9/12) of days. These findings are shown in Figure 2.

Data from LIAD were used to reveal activities that replaced planned physical activity or what activities led to unplanned MVPA. The analysis of time-use data from LIAD revealed several endeavors that replaced intended periods of physical activity. These activities were most often related to screen time (eg, using a computer, watching television, or reading), occurring on 56% (10/18) of days. Other categories included social endeavors (eg, spending time with friends or family), occurring on 22% (4/18) of days; sleep, occurring on 11% (2/18) of days; and miscellaneous activities (school and eating), occurring on 11% (2/18) of days. Conversely, for unplanned MVPA, most activities were related to personal care (eg, household chores and getting ready), occurring on 35% (6/17) of days, and active transportation (eg, walking to class or navigating urban areas), occurring on 35% (6/17) of days. Other activities included social endeavors, occurring on 12% (2/17) of days, and miscellaneous activities (physical therapy and classroom-based physical activity), occurring on 18% (3/17) of days. Among those who did not plan to be active and were not active, LIAD data revealed that these participants were mostly involved in work- or school-related activities during the day and screen time activities at night.

### Feasibility

Feasibility analyses indicated an average accelerometer wear time of 17 hours per day. In total, there was insufficient wear time for 8.3% (12/144) of days. All participants in the 1-day protocol had at least 10 hours of wear time per day, whereas 19% (3/16) of participants in the 3-day protocol and 31% (5/16) of participants in the 5-day protocol had invalid wear time for at least one day. This difference was not statistically significant (*P*=.054). When using the Google Calendar, 4% (2/48) of participants in the 1-day protocol and 4% (2/48) of participants in the 5-day protocol were missing calendar data. This difference was not statistically significant (*P*=.53). All participants, regardless of protocol length, recorded at least three activities in the LIAD app. When describing the difficulty of using LIAD, 73% (35/48) of participants mentioned the words *easy* or *simple*. Among those who reported some difficulty, 23% (3/13) of participants were from the 1-day protocol, 54% (7/13) of participants were from the 3-day protocol, and 23% (3/13) of participants were from the 5-day protocol. This difference was not statistically significant (*P*=.22). When using LIAD, 90% (43/48) of participants reported that most of their activities were recorded in real time. Among those who reported only recording some or few of their activities in real time, 40% (2/5) of participants were from the 1-day protocol and 60% (3/5) of participants were from the 3-day protocol. This difference was not statistically significant (*P*=.35). The most commonly reported barriers to record activities in real time included the challenge of remembering to record short activities, trying to start an activity when another is ongoing, and deciding which of the 12 categories to use (they had the option to add their own category). When asked about their participation, 96% (46/48) of participants were willing to enroll in a similar study again. The remaining 4% (2/48) of participants noted a difficulty in carrying a study phone along with a personal device as a barrier to further participation. Overall, 2869 activities were recorded in the LIAD app across 48 participants (mean 20, SD 9.1 activities per day; range 5-50).

## Discussion

### Principal Findings

Overall, we found fair congruence between plans to be active (or inactive) and physical activity behavior. However, the implementation rate of physical activity among those who planned to be active was only 40%, consistent with previous literature suggesting that there are additional factors that contribute to the relationship between physical activity plans and actual behavior (positive intenders). Intention as a proximal determinant of physical activity behavior is widely theorized, but evidence suggesting intention as a less robust predictor has prompted research into potential augmentations of the TPB model [47,48]. Specifically, dynamic changes in plans to be physically active in real-life settings are not captured well by the traditional TPB [18]. Here, we examined this aspect of the TPB by assessing the relationship between plans for physical activity and subsequent physical activity on a finer time scale (day level). To capture changes in intentions and plans in real time and the contextual factors that may influence this change, methods labeled under the *EMA* umbrella have been developed, assisted by advances in mobile phone technology [49]. The study of mobile phone technology as an innovative arm of EMA research has shown promise as a valid, feasible, and powerful tool across multiple populations [50,51].

This study adds unique information to help understand what occurs when plans to be physically active do not materialize. LIAD allows us to answer novel questions, such as those related to identifying replacement activities for planned physical activity and what activities occurred when participants did not plan to be active but were. When examining what activities were displacing physical activity in those periods, we found that most were related to screen time, specifically watching television or working on a computer. The relegation of physical activity in favor of sedentary, screen-related pursuits is consistent with the literature [52,53]. We also found that activities that resulted in MVPA but were not planned as times to be physically active generally included walking to class, navigating the campus, or doing household chores. These findings highlight the potential of using lifestyle choices and active transportation as tools to meet physical activity guidelines, especially when considering that one of the most highly cited reasons for inactivity among university students is the lack of time [8,54-56].

In recognizing these replacement pursuits, we improved our understanding of what factors determine the actual implementation of physical activity at the moment it is planned to occur. Among university students, this motivational flux may occur, in part, due to the accessibility of screen-related pursuits. Greater screen time has been found to be negatively associated with free-time physical activity [57]. Thus, promoting physical activity as part of a lifestyle routine may counteract the motivational flux seen in intention, removing the need to engage in periods of structured physical activity. Future studies could examine additional contextual factors such as location information, the influence of peers, and types of planned physical activities (eg, running in a park) to help inform the gap between plans to be physically active and actual physical activity and performance of a physical activity that was not planned. Furthermore, LIAD allows for an examination of what activities occurred over the course of the entire day and whether certain activities allow the flexibility to be active as planned, even when it was not during the planned time. Finally, future studies should consider collecting data from individuals over a longer period to understand intraindividual differences in implementation rates and factors that may predict any differences at the individual level.

Although LIAD and similar EMA data collection methods can be used as observational tools to shed light on changes in plans across different contexts and settings, they may also serve as a platform to *intervene* in real time. Mobile phone–based EMA interventions have previously been found to increase self-awareness of an individual’s mood and how they spend their time [49,58,59]. In exit surveys, multiple participants mentioned that LIAD made them more aware of how they spend their time, particularly as it relates to how busy they are. LIAD could be used in an intervention as part of a time management or awareness tool. In turn, this increase in self-awareness and mindfulness can prompt sustained behavioral changes in a range of health behaviors, including physical activity [49,60-62]. Finally, LIAD, in concert with other EMA apps, could provide feedback when people follow through on preferred planned behaviors or suggestions and encouragement when their plans fall through.

In this study, LIAD was found to be a feasible method for collecting time-use data in real time over a period of 1-5 days. Our feasibility analyses did not indicate any significant differences highlighted by protocol length, although differences in wear time approached significance. To reliably capture physical activity patterns, 3-5 days of accelerometer wear is recommended [29]. However, studies suggest that longer protocols result in greater sample loss, suggesting a need for researchers to find an appropriate balance [30,63]. With regard to LIAD, we found it to be an overall feasible and practical method of collecting time-use data in real time. Although we encountered few compliance issues, those who failed to record activity in real time were from the 1- or 3-day protocol. Previous literature has suggested that the effect of EMA protocol length on compliance, if not small, is somewhat random [64,65]. Rather, the frequency of EMA prompting is thought to have a more significant impact on compliance. Some studies have suggested that less frequent prompts (≤5 per day) are most beneficial to compliance [64,65]. However, there is limited quantitative evidence regarding the impacts of the frequency of EMA prompting on compliance and behavior change [66,67]. In this study, participants were not asked to respond to prompts but rather to record information when events occurred. This may serve as a good time to solicit information, as participants are not in the middle of an activity. This procedure resulted in a high number of recorded events, with up to 50 activities being recorded in a day and an average of 20 activities per day per participant. This, combined with a shorter protocol period (5 days or less), may be responsible for the high compliance with LIAD.

Given the reported difficulty of carrying 2 phones, compliance with reporting activities in real time may be improved if the LIAD app was used on a participant’s personal device. Future research should examine the differences between the inclusion of a study phone and the use of a personal device alone, as they relate to compliance and feasibility. We also did not prompt participants in an effort to boost compliance by not interrupting activities that were already occurring. Future research should continue to examine the effect of prompting through mobile devices on feasibility and compliance, including how many prompts are appropriate per day, how often participants should be prompted, and if prompting is more beneficial than allowing participants to record activities at their own volition [64-67]. A balance between recording events as they occur and infrequent prompting may show the greatest benefit to compliance and remembering to record activities in real time [65]. Participants also reported not knowing how to start an activity when another was ongoing as a barrier to record in real time. This may be improved by a more extensive explanation of the LIAD app during the intake meeting and by allowing participants to practice recording activities with the researcher going through various scenarios. Finally, participants noted difficulty in deciding which of the 12 categories to use. Future studies should make use of sequentially nested, fixed lists made possible by electronic time-use surveys, which could facilitate more detailed time-use information and may make the selection process easier for participants [28].

### Strengths and Limitations

This study has several strengths but also a few limitations. It is possible that our bout requirement for physical activity was too restrictive. Most participants recorded no MVPA, including some who planned to be active. Updated physical activity guidelines no longer require activity to be in bouts of 10 minutes or more. However, previous physical activity measurement research has largely used this 10-minute bout metric. Thus, we aimed to maintain consistency with this literature to facilitate comparison with the larger field. We also used wrist-worn accelerometers, although the most common placement for physical activity measurement is at the waist [29,68]. Recent advancements in triaxial accelerometry and algorithms have improved the accuracy of wrist-worn accelerometers, and using wrist placement has been shown to be less intrusive and increase compliance [69-71]. In this study, we used a well-known method for analyzing raw acceleration data and applying acceleration thresholds for MVPA [31-36]. We also may be limited by the small sample size, which might not be representative of the university student population. Finally, the participants were not required to record their planned activities in Google Calendar using the same categories as the LIAD app. It is possible that the classification of calendar activities into the same categories as the LIAD app by the research team could have introduced measurement errors. A strength of this study is its use of a time-use methodology to examine day-level plans with the possibility of identifying what activities replaced planned physical activity. In naming these physical activity replacements, we provided insight into factors driving the lack of execution of physical activity when it is planned. Other strengths included the ability to compare the feasibility of different lengths of protocol and the use of accelerometer data to objectively capture physical activity.

### Conclusions

In this study of university students, we found fair congruence between plans and physical activity behavior, as captured by a Google Calendar, a real-time time-use mobile phone app, and an accelerometer. A greater understanding of the factors that influence the implementation of physical activity plans may lead to more tailored and effective physical activity interventions that would increase physical activity levels. Most participants reported ease in using LIAD and had limited issues when recording their activities over the course of a day, suggesting that this method of data collection is feasible and practical for use in original research designs or interventions. The increasing popularity of mobile phones as an intervention tool for physical activity behavior indicates that these devices may be a very important entry point when intervening at the individual level [72,73]. With the ever-changing landscape of mobile phone technology and countless hours spent using these devices, future research should continue to examine mobile phones as tools for capturing time-use data, assessing physical activity–related indicators and as a means of intervening to change physical activity behavior.

## Appendix

Multimedia Appendix 1 Participants were instructed to record their activities on the home page of the app, which is located under the "My Activities" page.

Multimedia Appendix 2 Participants can start and stop activities.

Multimedia Appendix 3 A calendar in the upper right corner of the app allows participants to view the activities they have accumulated throughout the days in which they used the Life in a Day app.

## Abbreviations

EMA: ecological momentary assessment
LIAD: Life in a Day
MVPA: moderate-to-vigorous physical activity
PSID: Panel Study of Income Dynamics
TPB: theory of planned behavior
