# Supplementary figures and images for: Using a Mobile Phone App to Analyze the Relationship Between Planned and Performed Physical Activity in University Students: Observational Study
Source: JMIR Mhealth Uhealth. 2021 Apr 29;9(4):e17581. doi: 10.2196/17581 (PMC8120422; doi:10.2196/17581)

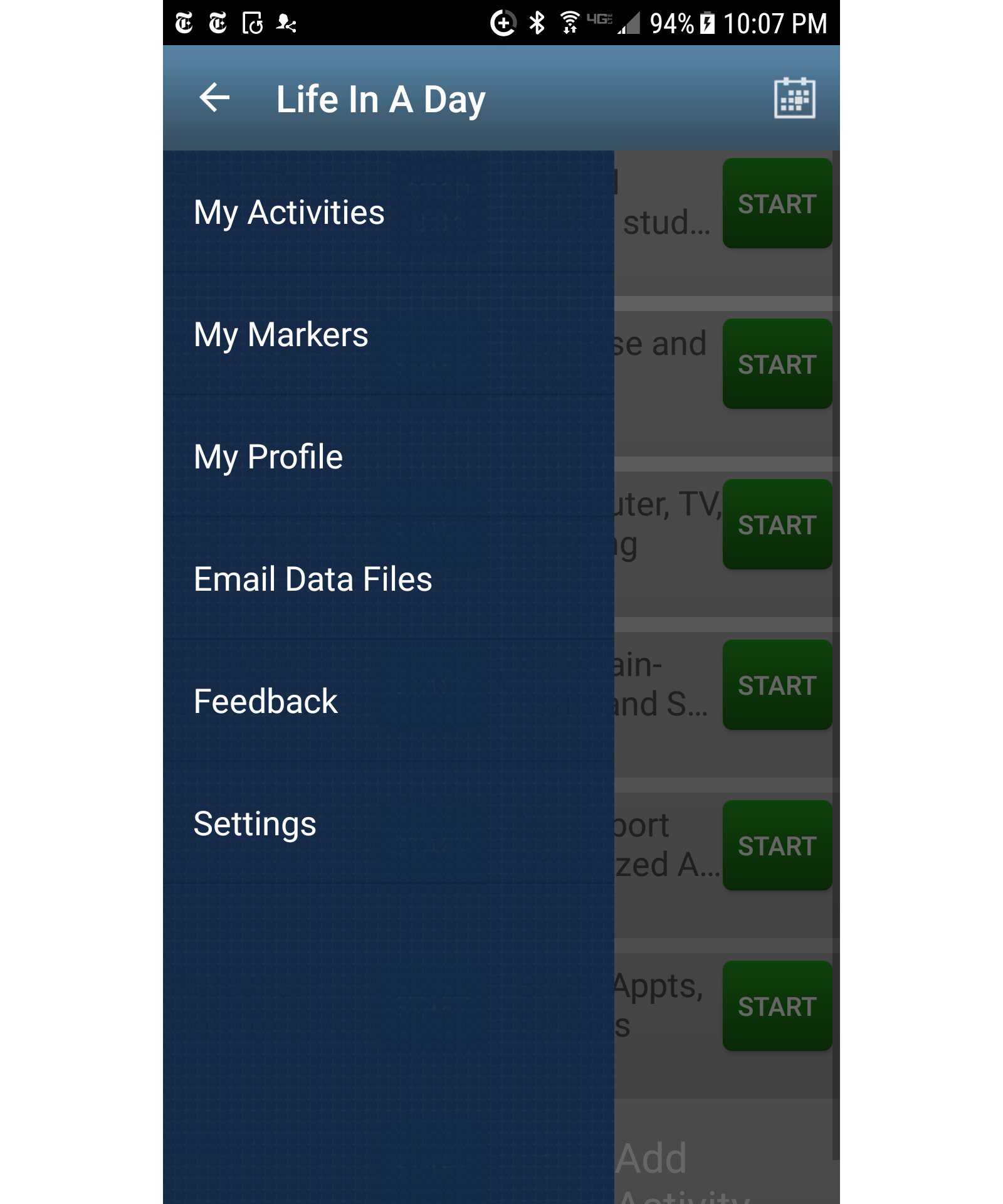

Supplement: Multimedia Appendix 1 [file mhealth_v9i4e17581_app1.png]

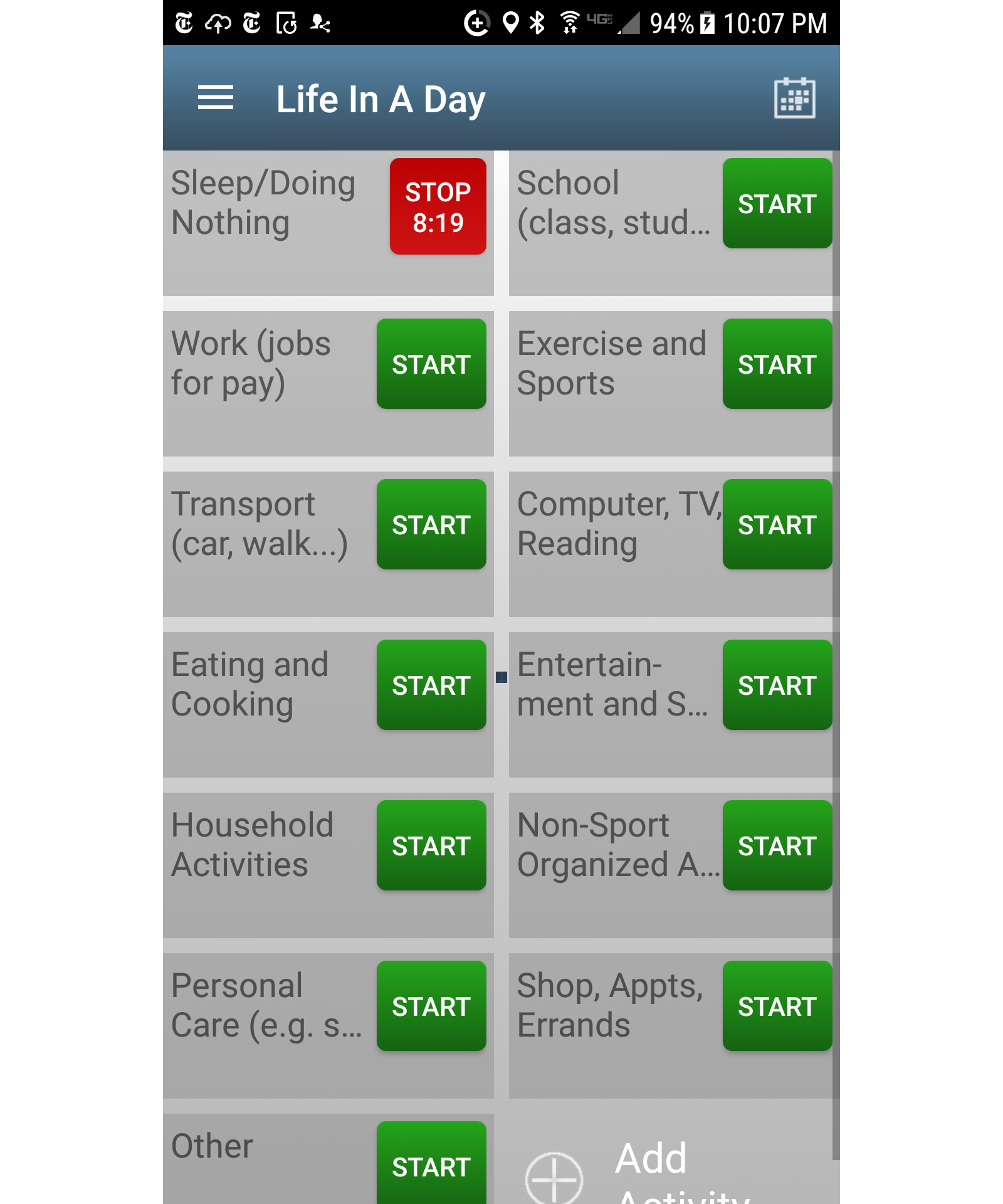

Supplement: Multimedia Appendix 2 [file mhealth_v9i4e17581_app2.png]

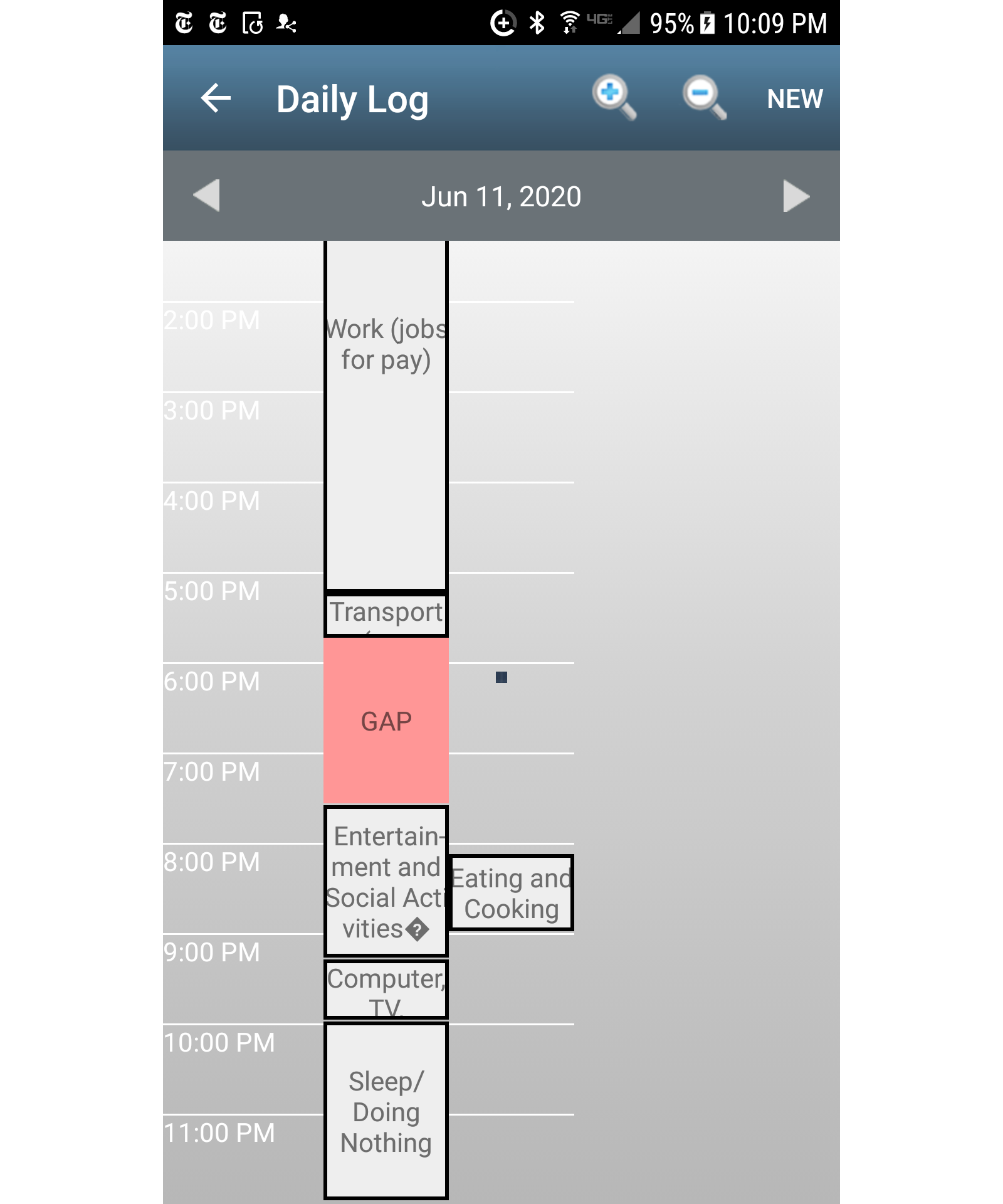

Supplement: Multimedia Appendix 3 [file mhealth_v9i4e17581_app3.png]
